# Potential Role of African Fermented Indigenous Vegetables in Maternal and Child Nutrition in Sub-Saharan Africa

**DOI:** 10.1155/2021/3400329

**Published:** 2021-12-15

**Authors:** Marie Lys Irakoze, Eliud N. Wafula, Eddy Owaga

**Affiliations:** ^1^Institute of Food Bioresources Technology, Dedan Kimathi University of Technology, Private Bag 10143 Dedan Kimathi, Nyeri, Kenya; ^2^Department of Physical and Biological Sciences, Bomet University College, P.O. Box 701-20400, Bomet, Kenya

## Abstract

Hunger and malnutrition continue to affect Africa especially the vulnerable children and women in reproductive age. However, Africa has indigenous foods and associated traditional technologies that can contribute to alleviation of hunger, malnutrition, and communicable and noncommunicable diseases. The importance of African indigenous vegetables is undeniable, only that they are season-linked and considered as “food for poor” despite their high nutritional contents. The utilization of African indigenous vegetables (AIVs) is hindered by postharvest losses and antinutrients affecting the bioavailability of nutrients. In Africa, fermentation is among the oldest food processing technologies with long history of safe use. Apart from extending shelf life and improving food organoleptic properties, fermentation of African indigenous vegetables (AIVs) is known to improve food nutritional values such as proteins, minerals, vitamins, and other beneficial phytochemicals. It can also increase bioavailability of various vitamins, minerals, and phytochemicals and increase synthesis of vital blood pressure regulators thus protecting against cardiovascular diseases and cancer and further helping fight certain malnutrition deficiencies. Some lactic acid bacteria (LAB) involved in food fermentation are known to produce exopolysaccharides with cholesterol-lowering, immunomodulator, antioxidant, and anticancer properties. Fermented foods (vegetables) are superior in quality and safety since most microorganisms involved in fermentation are good starter cultures that can inhibit the growth of foodborne pathogens and detoxify harmful compounds in foods. Thus, fermented foods can boost growth and well-being in children and women due to their higher nutritional contents. Therefore, fermentation of AIVs can contribute to the attainment of food and nutrition security especially among women and children who rely on these vegetables as a staple source of micronutrients and income. These benefits have a positive impact on the implementation of the second sustainable development goals and African Union agenda 2063. This review is aimed at shedding light on the potential of African fermented indigenous vegetables in combating maternal and child malnutrition in Sub-Sahara Africa.

## 1. Introduction

Currently, the world population is estimated at 7.8 billion with a projection of 9.8 billion in 2050 [1]. Further prediction shows that more than half of the world population will be living in Africa in 2050, with 2 in 5 being children [2].

The high population growth will put stress on food production which is even weak leaving many Africans food insecure especially for nourishing women and children who remain vulnerable and mostly found in rural areas where food is not sufficiently available or economic constraints preventing them from accessing sufficient nutrients [3]. Maternal and child malnutrition is a heavy burden on the health. It is therefore necessary to urgently act to ensure food and nutrition security [4].

To implement the second sustainable development goal (SDG 2) which stresses on the global access to safe, nutritious, and sufficient food and African Union Agenda 2063, Africa needs to do everything possible to provide sufficient and nutritious food to its growing population [2, 5, 6]. Particularly, regarding micronutrients, there is still a huge gap to fill; for example, Africa ranks number two in vitamin A deficiency [4, 7]. Around 60% of arable land is in Africa; unfortunately, more than a quarter of underfed people are found on this continent due to low agricultural and postharvest processing development among other causes [8]. In Sub-Sahara Africa (SSA), around 42% of children have symptoms of stunting which is the most noticeable form of malnutrition while women of reproductive age have shown a high prevalence of anemia [9].

AIVs are vegetables that originated or got established in Africa for many generations, and their leaves, young shoots, flowers, fruits, seeds, stems, tubers, or roots are consumed as vegetables [10, 11]. However, these vegetables also have the highest level of antinutrients especially polyphenols [12].

Since AIVs are adapted to the local climate and are readily available during the rainy season, they could contribute to the nutrient intake among the vulnerable people [13]. Therefore, there is need to process them with the purpose of keeping or increasing their nutrients, organoleptic properties, and storability [14, 15].

Among postharvest technologies, fermentation ranks among the oldest and most efficient because it requires less energy and materials while nutritionally enriching the food, increasing shelf life, reducing antinutrients, and increasing organoleptic properties [16, 17]. In Africa, fermentation is done at the household level and without uniformity of the products [3].

However, both fermented foods and indigenous vegetables have remained the foods of the poor which find almost no more place in the households' food preparations when lifestyle becomes better [18]. Fermented indigenous vegetables could play a potential role in ensuring food security in Africa [10, 11, 19], thus contributing to the alleviation of maternal and child low nutrient-related problems, hence safeguarding the whole family.

## 2. Methods

This review is aimed at shedding light on the potential of AIVs and fermentation of AIVs in combating maternal and child malnutrition in sub-Saharan Africa (SSA).

For this review, 11 articles, review papers, and reports ranging from 2010 to 2020 about the nutritional status of women and children in SSA were used. 56 on AIVs, their agricultural production, and their nutritional impact were collected, but 14 of them were excluded due to their irrelevance to the review. A total of 11 review articles on AIV fermentation were used, but 2 were excluded because they were too old, while 19 research articles and review papers on African fermented products were used in this manuscript.

## 3. Results

### 3.1. Maternal and Child Nutrition and Health Status in Sub-Saharan Africa

Stunting of children under 5 years and anemia among women of reproductive age prevail at an unacceptable level in Africa especially SSA [4, 20]. The low agricultural advances and natural and anthropological calamities have taken a lead in spreading hunger despite the second SDG and African Union Agenda 2063 which intends to eradicate hunger and undernutrition and ensure the provision of safe food to the people [3, 21]. SSA remains the one region of the earth with the highest prevalence of stunting, low birth weight, and mental retardation in children and anemia in pregnant and nonpregnant women and even with the general population having high micronutrient deficiency [7, 9].

In many communities of SSA, pregnant and lactating women are subjected to various food taboos which can result in malnutrition to them and their children [22]. Diarrhea remains among the most killer of children under the age of 5 especially in SSA [2, 3, 23]. Diarrhea can be due to low hygiene, but also malnutrition has a contribution in the frequent occurrence [16, 24]. Iron deficiency in women of reproductive age remains high in SSA despite iron supplement interventions [25–28]. It is believed that iron deficiency contributes to complicated pregnancies and iron deficiency in children which are prevalent in this region [29–31].

Iron, zinc, folate, and vitamin A deficiencies remain a big public health threat in Africa as affected women tend to have children with the same problems and consequences accumulate in the families if nothing is done to correct the situation [25, 32, 33]. There is still a need to improve maternal nutrition for better pregnancy outcomes [26, 28].

Even with all mitigation resolutions taken against micronutrient deficiency, in low-income countries (including SSA countries), 1.7% of deaths of children under the age of five are due to vitamin A deficiency [34]. Folate deficiency in pregnant and adolescent women is being combatted by food fortification and fermentation [31, 35].

Anemia like other malnutrition-related complications in women of reproductive age leads to low pregnancy outcomes and even mortality while stunting prevails in Africa with twenty-five countries having a highly unacceptable level of stunting (>30%) [20, 36]. This is because most women live in rural areas where they experience endless poverty, natural, and anthropological crises with all the responsibility to feed their children [3, 37]. Also, hunger and undernutrition are most prevalent among women and children [3, 37].

To alleviate the prevailing condition of micronutrient deficiency, many resolutions like food fortification and effective nutritional education need to be put in place [4]. However, AIV consumption and their good processing techniques are to be adopted among others for vulnerable households and the whole population to be able to meet their micronutrient needs [32]. Many AIVs were reported to contain micronutrients in sufficient amounts that they can alone help to attain the daily recommended intakes [10]. For instance, Moringa leaves can help to attain 80% of vitamin A daily requirements [30, 38].

### 3.2. Production and Diversity of AIVs in SSA

Indigenous vegetables are preferred over exotic ones as they are part of cultural heritage and medicinal or religious use [11, 19]. Africa must reinforce its food sovereignty by considering its indigenous food as one way to attain food security [10].

Production of AIVs is correlated to the cultural background, climate, and relief. However, cowpea (*Vigna unguiculata*), okra (*Abelmoschus esculentus*), African eggplants (*Solanum* spp.), some species of leaf amaranth (*Amaranthus* spp.), spider plant (*Cleome* spp.), some species of nightshades (*Solanum* spp.), and pumpkins (*Cucurbita* spp.) are more common throughout the continent [39].

Even if vegetable and fruit consumption in Africa remains low or depends on their seasonality, AIVs contribute a lot to nutritional intake and income generation [28, 40]. For instance, cowpea is rich in both *β*-carotene and iron, its production in Africa was estimated at around 6.7 metric tons in 2016, and many researches are ongoing to extend its shelf life and increase its overall acceptability [11, 41].

The diversity of indigenous vegetables is highly influenced by the climatic conditions in Africa; however, some are common in many parts of Africa, like African nightshades, African kale, and cowpeas [12, 15, 24].  Table 1 shows different vegetables and regions of Africa where they are commonly found.

In Kenya, the production of cowpea has steadily increased in this decade while in Rwanda, cassava leaf production and consumption in many parts of the country are almost in all households [42].

Indigenous vegetables are very nutritious and easy to find but are very perishable and available in seasons [10, 11, 38]. There is an imperative need to find a way of processing these vegetables increasing or maintaining their nutritional value, shelf life, and organoleptic properties [8, 43].

Fermentation is one of the promising technologies since it requires less sophisticated materials and energy [24, 41, 44, 45].

### 3.3. Nutritional Value and Challenges Facing Consumption of AIVs in SSA

#### 3.3.1. Nutritional and Phytochemical Composition of AIVs

AIVs are potential sources of various fibers, vitamins and minerals needed for healthy living. In many cases, AIVs are reported to be higher in micronutrients, proteins, and phytochemicals than the most exotic vegetables [33, 39, 50].  Table 2 illustrates the nutritional potential of some AIVs.

Some indigenous vegetables have a significantly recorded high protein, lipids, and mineral and vitamin content [12]. Like *Moringa oleifera* leaves have 6.6 mg/g of crude proteins and 1.5 mg/g of crude lipids, respectively, while *Amaranthus cruentus* has 5.3 mg/g and 3.0 mg/g of crude proteins and lipids, respectively [49].

Some AIVs are entitled to be the best in terms of micronutrient content, like in the case of *Morchella esculenta* which contains 1970 mg retinol equivalents/100 g of edible portion and 311 mg/100 g vitamin C while *Chenopodium album* has almost 6 mg/100 g iron, 18.5 mg/100 g zinc, 226 mg/100 g calcium, and 211 mg/100 g magnesium [15].

Most of AIVs have a high level of antioxidants, and they contribute significantly to antioxidant intake [56].

*Solanum scabrum* and *Abelmoschus caillei* are reported to contain 38.18 mg/g and 20.48 mg/g of proteins per edible portion, respectively, while *Solanum aethiopicum* has 624.54 *μ*g/g and 39.74 *μ*g/g of carotenoids and iron, respectively, which means that even a small portion of some indigenous vegetables like Moringa or black nightshade can give the body a significant amount of some nutrients like vitamin A [46, 49].

African cowpea leaves whether preserved or fresh are reported to have 0.25–36.55 mg *β*-carotene and 0.17–75.00 mg of iron 100 g dry weight, respectively [57]. Even if feeding on one type of vegetable cannot provide all the essential nutrients the body needs, diversifying the diet can help achieve a healthy life [5, 15].

Moreover, AIVs are known to contain remarkably high levels of phytochemicals in a wide range [56]. The phytochemicals in AIVs are attributed to health benefits and medicinal or religious use in different communities [57]. Phytochemicals are believed to prevent chronic diseases and metabolism disorders [53].

[58] reported the total extractable phenolic content in *A. dubius* to be 5.16 ± 0.12 mg/g DW and in *C. gynandra* to be 3.94 ± 0.09 mg/g DW while total flavonoid concentration in *A. dubius* to be 3.89 ± 0.28 mg/g DW, in *C. gynandra* to be 2.19 ± 0.11 mg/g DW, and in *C. maxima* to be 1.55 ± 0.04 mg/g dry weight. Though some antinutrients (like saponin, phytates, and tannins) are found in AIVs, their wide range of dietary phytochemicals is more complex from phenolic compounds, flavonoids, carotenoids, and glucosinolates [58, 59]

This phytochemical renders AIVs excellent free radical scavengers, such as *Nasturtium aquatica*, *Urtica dioica*, and *Xanthosoma mafaffa* [54].

#### 3.3.2. Benefits of AIVs in Maternal and Child Nutrition

The importance of AIVs on the food security in SSA was reported by [9, 39]. It is expected that the promotion of agriculture and consumption of AIVs will help in fighting hunger in both rural and urban households [32, 60].

To fight children's malnutrition, there is a need for food system which avails safe, affordable, nutritious, and sustainable food [55]. This promise is held by different food systems and foods including AIVs.

The AIVs are available and can be preserved to be used in lean season, and their nutritional and phytochemical content is so excellent that they can help maternal and child nutrition meet if promoted and processed [28, 41, 58].

Table 2 gives an insight into some nutritionally rich AIVs. Since many AIVs are reported to contain a tremendous amount of vitamins, minerals, and some other nutrients like proteins, lipids, and dietary fibers, they can help in malnutrition mitigation of women and children in SSA [28, 39].

The high vitamin A content in AIVs can help to alleviate death or other metabolic complications related to vitamin A deficiency [7, 11, 47]. Anemia due to iron or folate can be mitigated by increased consumption of AIVs [7, 10].

Also, metabolic disorders or lifestyle diseases can be prevented by the healthy phytochemicals found in AIVs allowing maternal and child health to be protected [58].

Moreover, meeting nutritional requirements can help the family especially maternal and child health, protecting them from malnutrition, complications related to low diet, retarded growth, and mortality [27, 32, 60]. Although these vegetables are not a hundred percent to alleviate poverty, their contribution is significant; their contribution to micronutrient intake can play a big role in fighting hidden hunger in SSA [38, 60].

#### 3.3.3. Socioeconomic Value of AIVs

Indigenous vegetables can help families to attain sufficient nutrient requirements while also helping them to generate income, leading to poverty reduction [19, 38]. Mainly, women are involved in farming and selling vegetables to raise their financial ability thus helping them to get more buying power to afford even other commodities [12].

Farming and other activities related to the indigenous vegetable can employ women or give them income which increases their purchasing power, especially the ability to buy more foods to meet the nutritional requirement [24, 51]. Ref. [12, 39] noted the tremendous economic importance of AIVs in the communities where they were exploited commercially.

Since these vegetables are associated with customs and traditional cultures, their agriculture and consumption can help in maintaining traditions [53, 57]. AIVs require less agricultural inputs and can be collected from the wild, which can be helpful towards ensuring that adequate access to food by everyone at home [30]. Also, it is worth noting that AIVs are prepared in a variety of ways even in one community, thus allowing them to consume enough nutrients from available AIVs even during distress time [30]. Engaging in AIV agricultural business has the potential of increasing income generation to women resulting in their empowerment [19, 49]. This women empowerment and economic growth are strongly related to better child nutrition [46].

#### 3.3.4. Challenges Contributing to Limited Consumption of AIVs in SSA

Consumption of AIVs has decreased even if they are believed to contain many nutrients and healthy phytochemicals [10, 13, 52]. This decline can be associated with the fact that they are considered to be for the poor man, and the agricultural shift towards exotic vegetables has lowered their cultivation leaving them to be used only during distress times even if their potential to help achieve the daily nutritional requirements remains known [30, 47, 61]. [46] noted that there is lack of nutritional awareness of the AIVs and that it is needed to disseminate the importance of AIVs in addressing malnutrition and hunger.

Some of the factors associated with low consumption of AIVs include high antinutrient content, their complicated preparation methods, high perishability and seasonality, and loss of indigenous knowledge trickle down to subsequent generations [57, 61–63]. For example, postharvest losses of cowpea in East Africa are estimated at around 30-40% of the production; this can be one of the challenges which are hindering the consumption of these AIVs [56].

### 3.4. Potential Benefits of Fermented AIVs on Nutrition

#### 3.4.1. Fermentation of Vegetables

Fermentation is partial oxidation of carbohydrates into ethanol or organic acids and CO_2_, carried by lactic acid bacteria (LAB), yeasts, and some mold species, it is among the oldest human technologies, and its knowledge is a legacy of generation to generation [8, 64–66].

Fermentation of vegetables is mainly lactic acid fermentation and alkaline fermentation (which involves degradation of proteins and polysaccharides into smaller readily absorbed molecules) [44, 65]. In Africa, vegetables are lactic acid-fermented to produce plant proteins and condiments [67].  Table 3 gives an example of known fermented AIVs in Africa. Traditional fermentation knowledge coupled with advanced food technologies is promising a better future of providing safe and healthy foods to the world's growing population [22].

From a global perspective, fermented foods are inherent components of diets as they usually have characteristic properties like flavor, aroma, appearance, or consistency which increase their appeal [22, 68].

Fermented vegetable products are still attracting more research because of their relatively longer shelf life, nutritional benefits, and safety [13, 68, 69]. Fermented foods are associated with several social and cultural aspects of different human communities due to their high nutritional value [43, 67].

Fermentation has been associated with preservation, detoxification, and creation of desirable organoleptic properties [43, 70]. Some of the health benefits of fermented foods include increased bioavailability of vitamin B_2_ (riboflavin), vitamin B_9_ (folate), vitamin B_12_, and vitamin K. In addition, increased synthesis of melatonin and Gaba regulates blood pressure and protects against cardiovascular disease and cancer. The produced exopolysaccharides have cholesterol-lowering, immunomodulator, antioxidant, and anticancer properties and a variety of bioactive peptides that possess antihypertensive, anticancer, anti-inflammatory, antidiabetic, antimicrobial, antiadipogenic, antimutagenic, antithrombotic, and antiatherogenic properties [22, 63].

#### 3.4.2. Fermentation of Vegetables in Africa

In Africa, fermentation of vegetables is mainly lactic acid fermentation and alkaline fermentation (which involves degradation of proteins and polysaccharides into smaller readily absorbed molecules) [11, 65]. Fermentation of AIVs is mainly to improve the sensory properties and extend the shelf life [71].  Table 3 gives examples of known fermented AIVs in Africa. [71, 72] observed that since most of the fermented vegetables and condiments in SSA are used in sauce preparations, they provide proteins and vitamins in addition to flavoring the sauces.

Traditional fermentation knowledge coupled with advanced food technologies is promising a better future to feed safe and healthy foods to the world's growing population as the awareness of fermented food's health benefits is sharply rising [22]. Although there is still less documentation of fermented AIVs, the processes and microorganisms involved. Researchers are striving to show the richness of these foods. [24] observed that fermented cowpea was high in nutrients and could last longer. Fermented African black nightshade was observed to contain more water-soluble vitamins than solar-dried or cooked counterparts [37].

Fermentation of AIVs is promising to continue to enrich African food systems. Africans know about fighting certain malnutrition conditions like vitamin deficiency using fermented products as fermentation increases bioavailability of micronutrient [13, 18, 73].

#### 3.4.3. Benefits of Fermented AIVs

Fermentation of AIVs can help in increasing nutritional value while detoxifying the toxic one [11]. Since many indigenous vegetables are seasonal, fermenting them can increase their shelf life, thus reducing postharvest losses and deficiency in lean season [22, 74].

Microbiologically and chemically, fermentation ensures the safety of the food by overcompeting bad microorganisms by producing antimicrobial chemicals such as bacteriocins, organic acids, diacetyl, and CO_2_ [22, 63]. The lowering of the pH inhibits the growth of harmful microorganisms while the overall fermentation process results in the degradation of toxic phytochemicals into less harmful or healthy products [22, 72]. Since fermentation is a less energy-consuming and less sophisticated technology, it can be made at the household level to local commercial scales [48, 73, 75].

Indigenous vegetables and indigenous fermented foods play a huge role in household food security and safety especially important for maternal and child nutrition [14, 18, 44]. Many indigenous vegetables are reported to have a high content of phytochemicals or nutrients which are bound by antinutrients; fermentation can avail the nutrients while detoxifying the food allowing consumers to absorb nutrients [11, 64]. Even if AIVs are famous for their high content of minerals, their high content of antinutrients like phytates, tannins, and oxalates makes the minerals less biologically available [66].

Fermentation reactions can break the bonds between the minerals and the bound antinutrients, thus increasing their bioavailability [44]. An increase in free amino acids in fermented products has been observed which makes fermented products readily absorbable in the intestines [24]. Fermented vegetables like other indigenous foods play important role in the food security of communities as they increase palatability, shelf life, and safety [11, 41, 48, 73].  Table 2 discusses some fermented indigenous vegetables in Africa and their uses in their respective region of origin.

During the investigation of the health benefits of fermented products, [22, 44, 76] underlined the health benefits of lactic acid fermentation products ranging from prevention of diarrhea through blood pressure lowering to anticancer properties of vitamins, minerals, and bioactive compounds produced during the process. Fermentation of different AIVs like the African locust beans degrades some proteins into smaller peptides hence increasing their digestibility [8, 11, 67, 71].

AIVs are fermented to increase protection against gastrointestinal diseases, heart diseases, and diabetes. Mostly in SSA, fermented AIVs are associated with health benefits in addition to the nutrients, palatability, and shelf life [67]. Most of the children particularly during weaning fed on fermented foods ranging from sour milk, sour gruel, and porridges or sauces [76, 77]. These foods have probiotic properties and higher nutritional content than their conventional relatives. They are believed to boost growth in children and well-being in the general population [40]. Locals appreciate these products because of their ability to fight both diarrhea and constipation, common complications in children [40, 77]. These lactic acid-fermented products have an increased level of phenolic compounds [44, 78].

The wide range of health benefits attributed to fermented products including the increased digestibility, reduced food intolerance and allergic reaction, or increased bioavailability of nutrients and bioactive compounds which have been noted [63, 73]. It has been noted that the lowering of pH in fermented foods prevents the growth of harmful microorganisms and prevents them to produce their toxic compounds [71].

Increased shelf life of fermented products can help women to be sure of food security in their house even during lean seasons, thus helping them to ensure intake of essential nutrients and preventing under-nutrition-related diseases [33, 66]. Also fermenting indigenous vegetables can be a source of income to women who are mainly the ones to take care of food provisions and preparations [14, 18]. With a source of income, women can be able to buy even the food they do not grow, allowing them to meet necessary nutritional requirements and to meet the necessary hygiene needed for a healthy life [19, 44, 75].

#### 3.4.4. Role of Lactic Acid Fermentation on Nutrition and Health of Child and Women

Lactic acid fermentation is the most common type of vegetable fermentation due to its numerous attributes ranging from enhancing food safety, quality, and biopreservation and removing antinutrients, to improving the health of the consumer [67, 70]. This fermentation produces lactic acid and bacteriocins among other products; these metabolites are useful in lowering the pH and suppressing the growth and toxin production of other microorganisms, particularly the pathogens and spoilage microorganisms; hence, the products of this process are microbiologically safe [26, 66, 69]. Lowering the pH of the product prevents microorganisms like *Clostridium botulinum* to produce their toxins in this case botulin [73]. Enhancing flavor and other organoleptic properties is a characteristic of lactic acid bacteria [68].

Lactic acid bacteria produce several compounds as byproducts of lactic acid fermentation in which vitamin B complex and K are among the secondary metabolites [63, 79]. Complex biomolecules are degraded into many small and readily absorbable molecules during lactic acid fermentation, and it helps people with digestive complications like lactose intolerance and people with allergies [22]. Some lactic acid strains are believed to remove or reduce mycotoxins from fermented foods [26].

[44] suggested that lactic acid bacteria can digest proteins or other complex molecules like polysaccharides increasing the bioavailability of nutrients. Lactic acid-fermented foods are nutritionally good and can contribute to the alleviation of hidden hunger [18, 28, 43]. Mostly, fermentation of AIVs intends to harness the higher proteins and other bioactive compounds [29]. It has been noted that up to 74% of Africans believe in good outcomes of feeding children from 6 months on fermented foods [77]. Fermented AIVs are liked as cheap sources of proteins, B complex, minerals, and more bioactive compounds [29]

Lactic acid bacteria notably *Lactobacillus fermentum*, *Lactococcus casei*, *L. plantarum*, and *L. reuteri* are probiotics found in fermented foods. They are known to increase phenolic compounds while reducing oxalates and tannins [44, 78]. LAB are known to regulate natural and acquired immune responses [69, 79]. Thus, lactic acid-fermented foods give this advantage to the body.

In fermented AIVs like other fermented products, the nutritional content is higher than in other food preparation methods. Proteins, vitamins, minerals, and phytochemicals have been reported to be increased or at least conserved more than other food preparation techniques [17, 24]. Fermented vegetables are known to prevent food poisoning and can help maintain the health of children and mothers [44].

The lactic acid bacteria involved in fermented products colonize the gastrointestinal tract of the host and help them to fight bad microbes by their antibacterial activities [69, 79]. In weaning, children fed on fermented products which are believed to protect them from diarrhea and provide them with essential nutrients needed for growth [29, 44, 77].

Different products are made using LAB from sour milk and cheese through fermented vegetables to sour alcoholic drinks, and these diverse products have shaped the civilizations and are expected to continue helping human communities to strive [75, 80]. Biopreservation and enhanced sensory properties insured by lactic acid fermentation of AIVs will allow food security to be met hence helping maternal and child nutrition in the SSA [44, 69, 75]. Some products are believed to relieve or prevent dental carries which are common in pregnant and breastfeeding women. There has been an observation of *Lactobacillus* and *Bifidobacterium* species (commonly found in African fermented products) helping with preventing dental caries [69, 77].

As far as todays' trend in healthy foods remains, fermentation of AIVs will be a source of income that can help women's economic growth, which can help them achieve secured, safe, and sustainable nutrition in their households [35, 40]. And ensuring the continuation of utilization AIVs and fermenting them will underline the food sovereignty and security of Africa [11].

## 4. Current and Future Challenges

To alleviate hunger in its all manifestations whether stunting, wasting, or mental retardation of African children and anemia and micronutrient deficiency in African women of reproductive age, indigenous foods and indigenous food technologies are among the promising sustainable treatments.

Currently, little is documented about indigenous vegetables and indigenous technologies especially fermentation of vegetables in Africa. There is lack of elaborate fermentation technology in Africa. Each region has its way of fermenting its products which makes it difficult to adopt the fermentation on a continent level. The diversity of fermentation processes is influenced by the end product desired characteristics, climate, and microorganisms involved.

With the loss of generation trickle-down of knowledge sharing, more is discussed about optimization of the process. Inconvenience and poor attitude towards AIVs and indigenous fermented products by urban dwellers or economically improved people remain a challenge for this development. Therefore, research is needed to shed light on indigenous foods and food technologies and their potential in combating rampant malnutrition, child, and maternal health problems in SSA. This will allow the African continent to realize her aspirations as envisaged in agenda 2063.

## Figures and Tables

**Table 1:** Diversity of African indigenous vegetables [10, 13, 15, 21, 30, 38, 39, 42, 46–55].

Vegetable	Consumed parts	Scientific name	Region where they are consumed
Black African nightshades	Leaves and young shoots	*Solanum nigrum* and *S. scabrum*	Tropical Africa
African spider plant	Leaves and young shoots	*Cleome gynandra*	Tropical Africa and semiarid regions
African eggplant	Berries	*Solanum aethiopicum*	Central, East tropical, and Eastern Africa
Jute mallow	Leaves and young shoots	*Corchorus olitorius*	East Africa
Amaranthus	Leaves and young shoots	*Amaranthus cruentus*	Tropical and semiarid Africa
Roselle hibiscus	Flowers and seeds	*Hibiscus sabdariffa*	Eastern Africa
Cowpeas	Leaves and young shoots	*Vigna unguiculata*	Tropical Africa
Baobab	Leaves, young shoots, and fruits	*Adansonia digitata*	West, Central, and East tropical Africa
Moringa	Leaves, bark, roots, and fruits	*Moringa oleifera*	West, Central, and East tropical Africa
Cassava leaves	Leaves	*Manihot esculenta*	West, Central, and East tropical Africa
Plumed cockscomb or silver cock's comb	Flower and leaves	*Celosia argentea*	West, Central, and East tropical Africa
Mkula	Leaves and young shoots	*Pterocarpus mildbraedii*	West tropical and East tropical Africa
Sickle pod	Leaves	*Senna obtusifolia*	Eastern Africa/semiarid regions
Watercress	Leaves and young shoots	*Rorippa madagascariensis*	Tropical Africa
Burweed	Leaves	*Triumfetta annua*	All over tropical Africa
Kenaf	Young leaves and shoots	*Hibiscus cannabinus*	Tropical regions
Sorrel	Young shoots	*Oxalis*, *Rumex acetosa*	Tropical regions
		*Cucumis africanus*	Southern Africa
Ethiopian kale	Leaves	*Brassica carinata*	Northern and Eastern Africa
Sweet potato leaves	Leaves	*Ipomea batata*	Tropical Africa
Beans leaves	Leaves	*Phaseolus vulgaris*	Tropical Africa
Taro root leaves	Leaves	*Colocasia esculenta*	Tropical Africa
Blackjack	Young shoots	*Bidens pilosa*	Tropical Africa
Chayote	Fruit	*Sechium edule*	Tropical Africa
Chicken spike	Young leaves and shoots	*Sphenoclea zeylanica*	Africa
Turkey berries	Berries	*Solanum torvum*	Tropical Africa
Okra	Pod	*Abelmoschus esculentus* & *A. caillei*	Tropical Africa and semiarid regions
Sesame	Seeds	*Sesamum indicum*	Tropical, subtropical, and temperate Africa
Pumpkin leaves	Leaves	*Cucurbita moschata* & *C. maxima*	Africa
Bacon weed	Leaves	*Chenopodium album*	Tropical Africa
Groundnut	Beans	*Arachis hypogea*	Tropical Africa
Watercress	Leaves and young shoots	*Nasturtium aquatica*	Tropical Africa
Stinging nettle	Leaves and young shoots	*Urtica dioica*	Tropical and arid regions of Africa
Arrow leaf/elephant's ear	Leaves	*Xanthosoma mafaffa*	Tropical Africa
African foxglove	Leaves	*Ceratotheca triloba*	Southern Africa
Yellow Justicia	Leaves	*Justicia flava*	Tropical and Southern Africa
Waterleaf	Leaves	*Talinum triangulare*	West tropical Africa
Slender leaf	Leaves	*Crotalaria brevidens*	Tropical Africa and cultivated in temperate regions
Gallant soldier	Leaves	*Galinsoga parviflora*	Tropical Africa
Bitter leaf	Leaves	*Vernonia amygdalina*	Tropical and Southern Africa

**Table 2:** Micronutrient content of edible part of some indigenous African vegetables.

Nutrients	Vegetables	Range content	Reference	Recommended daily safe intake	
*Β*-carotene	*Moringa oleifera* (leaves)	19.74 mg/100 g DW		Children (0-7 years)	Pregnant and lactating women
	*Manihot esculenta* (leaves)	16.87 mg/100 g DW			
	*Solanum nigrum* (leaves)	1070 mg/100 g DW			
	*Ipomea batatas* (leaves)	980 mg/100 g DW			
	*Bidens pilosa* (leaves)	985 mg/100 g DW			
			[12, 15, 33, 46, 53, 57]	375-500 *μ*g RE	800-850 *μ*g RE
Vitamin C	*Moringa stenopetala* (leaves)	400 mg/100 g DW	[15, 33, 39, 42, 57]	35-30 mg	55-70 mg
	*Manihot esculenta* (leaves)	387 mg/100 g DW			
	*Moringa oleifera* (leaves)	274 mg/100 g DW			
	*Ipomea batata* (leaves)	70 mg/100 g DW			
Vitamin E	*Moringa stenopetala* (leaves)	17.72 mg/100 g DW	[33, 42]	4-5 mg	15-19 mg
	*Moringa oleifera* (leaves)	13.44 mg/100 g DW			
	*Manihot esculenta*(leaves)	12.77 mg/100 g DW			
Folates	*Cleome gynandra* (leaves)	198 *μ*g/100 g DW	[15, 33, 39, 57]	80-300 *μ*g	500-600 *μ*g
	*Celosia argentea* (leaves)	159 *μ*g/100 g DW			
	*Solanum nigrum* (leaves)	404 *μ*g/100 g DW			
	*Ipomea batata* (leaves)	80 *μ*g/100 g DW			
	*Bidens pilosa* (leaves)	315 *μ*g/100 g DW			
Calcium	*Moringa stenopetala* (leaves)	711 mg/100 g DW	[15, 33, 46, 57]	300-800 mg	1000-1200 mg
	*Senna obtusifolia* (leaves)	589 mg/100 g DW			
	*Moringa oleifera* (leaves)	584 mg/100 g DW			
Iron	*Triumfetta annua* (leaves)	29.2 mg/100 g DW	[33, 46, 49, 53]	3.9-6.2 mg for 15% bioavailability	10.0-21.9 mg for 15% bioavailability
	*Hibiscus cannabinus* (leaves)	12.1 mg/100 g DW			
	*Cucumis africanus* (leaves)	12 mg/100 g DW			
Zinc	*Pterocarpus mildbraedii* (leaves)	3.1 mg/100 g DW	[33, 46, 53, 57]	1.1-3.3 mg	3.4-6.0 mg
	*Moringa oleifera* (leaves)	2.8 mg/100 g DW			
	*Manihot esculenta* (leaves)	1.79 mg/100 g DW			
Antioxidants	*Adansonia digitata* (leaves)	13,506 TE/g	[33, 39, 50, 57, 59]		
	*Rorippa madagascariensis* (leaves)	12,839 TE/g7527 TE/g			
	*Manihot esculenta* (leaves)				
Total phenolics	*Rorippa madagascariensis* (leaves)	2189 mg/100 g DW	[33, 39, 42, 50, 57, 59]		
	*Manihot esculenta* (leaves)	1863 mg/100 g DW			
	*Adansonia digitata* (leaves)	2140 mg/100 g DW			

TE/g represents mM of ascorbic acid equivalent per g of dry mass.

**Table 3:** Fermented vegetables of Africa.

Products	Plant	Latinized name	Use	Reference
Kawal	Sickle pod	*Cassia obtusifolia*	Meat substitute	[11, 24]
Dawadawa/Iru/Sumbala	African locust beans	*Parkia biglobosa*	Condiment	[11, 63]
Ntoba mbodi	Cassava leaves	*Manihot esculenta*	Condiment	[11, 22]
Fermented cowpea leaves (Kunde)	Cowpea leaves	*Vigna unguiculata*	Condiment	[11, 22, 63]
Fermented Ethiopian kale	African kale	*Brassica carinata*	Condiment	[11, 63]
Fermented African black nightshade leaves	African black nightshade	*Solanum scabrum*	Condiment	[11, 17, 63]
Okpehe	Iron tree	*Prosopis africana*	Condiment	[11, 63]
Maari	Baobab seeds	*Adansonia digitata*	Condiment	[11, 63]
Bikalga/Furundu	Oiselle	*Hibiscus sabdariffa*	Condiment	[11, 63]
Ugba	African oil bean seed	*Pentaclethra macrophylla*	Condiment	[11, 63]
Sigda	Sesame oil seeds press cake	Sesamum indicum	Meat substitute	[11]
Teshnuti	Okra seeds	*Abelmoschus esculentus*	Meat substitute	[11]
Kirjigil	Sesame seeds, pumpkin seeds, cowpea	*Sesamum indicum*, *Cucurbita spp.*, *Vigna unguiculata*	Meat substitute	[11]
Rob-heb	Watermelon seeds	*Citrullus lanatus*	Condiment	[11]
Urn-Zummatah	Watermelon juice	*Citrullus lanatus*	Beverage	[11]

## Data Availability

The reference data used to support the findings of this study are included within the article.

## References

[1] UN (2021). *Population | United Nations*.

[2] UNDP (2012). The nutrition challenge in sub-Saharan Africa (issue January). by Jessica, F. on behalf of UNDP. http://www.undp.org.

[3] FAO (2020). 2019 Africa regional overview of food security and nutrition. *2019 Africa Regional Overview of Food Security and Nutrition*.

[4] Harika R., Faber M., Samuel F., Kimiywe J., Mulugeta A., Eilander A. (2017). Micronutrient status and dietary intake of iron, vitamin a, iodine, folate and zinc in women of reproductive age and pregnant women in Ethiopia, Kenya, Nigeria and South Africa: a systematic review of data from 2005 to 2015. *MDPI*.

[5] Amaral G., Bushee J., Cordani U. G. (2013). Patterns and determinants of fruit and vegetable consumption in sub-Sahara Africa: a multicountry comparison. *Journal of Petrology*.

[6] UNICEF (2020). UNICEF. Diarrhoea in children under 5 years old. https://data.unicef.org/topic/child-health/diarrhoeal-disease/.

[7] Hoffman D. J., Merchant E., Byrnes D. R., Simon J. E. (2018). Preventing micronutrient deficiencies using African indigenous vegetables in Kenya and Zambia. *Sight and life*.

[8] Ekhuya N. A., Wesonga J. M., Abukutsa-Onyango M. O. (2018). Production, processing and storage techniques of African nightshade (solanum spp.) seeds and their correlations with farmers’ characteristics in western Kenya. *African journal of food, agriculture, nutrition and development*.

[9] Githanga D., Awiti A., Were F., Ngwiri T., Nyarko M. Y., Shellack N. (2019). A consensus on malnutrition in Africa: a report from the micronutrient deficiency awareness forum (Nairobi 2017). *African Journal of Food, Agriculture, Nutrition and Development*.

[10] Ogundiran A. (2019). Food security, food sovereignty, and indigenous knowledge. *African Archaeological Review*.

[11] Oguntoyinbo F. A., Fusco V., Cho G. S. (2016). Produce from Africa’s gardens: potential for leafy vegetable and fruit fermentations. *Frontiers in Microbiology*.

[12] Oladele O. I. (2011). Contribution of indigenous vegetables and fruits to poverty alleviation in Oyo State, Nigeria. *Journal of Human Ecology*.

[13] Gido E. O., Ayuya O. I., Owuor G., Bokelmann W. (2017). Consumption intensity of leafy African indigenous vegetables: towards enhancing nutritional security in rural and urban dwellers in Kenya. *Agricultural and food economics*.

[14] Ba D. M., Ssentongo P., Kjerulff K. H. (2019). Adherence to iron supplementation in 22 sub-Saharan African countries and associated factors among pregnant women: a large population-based study. *Nutritional Epidemiology and Public Health*.

[15] Uusiku N. P., Oelofse A., Duodu K. G., Bester M. J., Faber M. (2010). Nutritional value of leafy vegetables of sub-Saharan Africa and their potential contribution to human health: a review. *Journal of Food Composition and Analysis*.

[16] Carvajal-Vélez L., Amouzou A., Perin J. (2016). Diarrhea management in children under five in sub-Saharan Africa: does the source of care matter? A countdown analysis. *BMC Public Health*.

[17] Wafula E. N., Franz C. M., Rohn S., Huch M., Mathara J. M., Trierweiler B. (2016). Fermentation of African leafy vegetables to lower post-harvest losses, maintain quality and increase product safety. *African Journal of Horticultural Science*.

[18] Gadaga T., Lehohla M., Ntuli V. (2020). Traditional fermented foods of Lesotho. *Journal of Microbiology, Biotechnology and Food Sciences*.

[19] Odongo G. A., Schlotz N., Baldermann S. (2018). African nightshade (Solanum scabrum mill.): impact of cultivation and plant processing on its health promoting potential as determined in a human liver cell model. *Nutrients*.

[20] Binagwaho A., Rukundo A., Powers S. (2020). Trends in burden and risk factors associated with childhood stunting in Rwanda from 2000 to 2015: policy and program implications. *BMC Public Health*.

[21] AU (2015). *The African Union Commission Agenda 2063 Framework Document*.

[22] Soni S., Dey G. (2014). Perspectives on global fermented foods. *British Food Journal*.

[23] FAO/WHO (1998). *Vitamin and Mineral Requirements in Human Nutrition. Second Edition*.

[24] Owade J. O., Abong’ G., Okoth M., Mwang’ombe A. W. (2020). A review of the contribution of cowpea leaves to food and nutrition security in East Africa. *Food Science and Nutrition*.

[25] Bationo F., Songré-Ouattara L. T., Hama-Ba F. (2020). Folate status of women and children in Africa– current situation and improvement strategies. *Food Reviews International*.

[26] Mgamb E., Gura Z., Wanzala P. (2017). Folate deficiency and utilization of folic acid fortified flour among pregnant women attending antenatal clinic at Pumwani maternity hospital, Kenya. *Pan African Medical Journal*.

[27] Moschovis P. P., Wiens M. O., Arlington L. (2018). Individual, maternal and household risk factors for anaemia among young children in sub-Saharan Africa: a cross-sectional study. *BMJ Open*.

[28] Nnam N. M. (2015). Improving maternal nutrition for better pregnancy outcomes. *Proceedings of the Nutrition Society*.

[29] Ayoade O. N., Chimezie O. P. (2019). African fermented food condiments: microbiology impacts on their nutritional values. *Frontiers and New Trends in the Science of Fermented Food and Beverages*.

[30] Mbhenyane X. G. (2017). Indigenous foods and their contribution to nutrient requirements. *South African Journal of Clinical Nutrition*.

[31] Mora-Villalobos J. A., Montero- Zamora J., Barboza N. (2020). Multi-product lactic acid bacteria fermentations: a review. *Fermentation*.

[32] Jones R., Haardörfer R., Ramakrishnan U., Yount K. M., Miedema S., Girard A. W. (2019). Women's empowerment and child nutrition: the role of intrinsic agency. *Health*.

[33] Ngeywa T. T. (2016). The role of African indigenous leafy vegetables in immune boosting by Teresa Ngeywa Tumwet. *A Thesis Submitted in Fulfillment for the Degree of Doctor of Philosophy in Applied Human Nutrition Department of Food Science*.

[34] Stevens G. A., Bennett J. E., Hennocq Q. (2015). Trends and mortality effects of vitamin a deficiency in children in 138 low- income and middle-income countries between 1991 and 2013: a pooled analysis of population-based surveys. *The Lancet Global Health*.

[35] Bintsis T. (2018). Lactic acid bacteria: their applications in foods. *Journal Of Bacteriology & Mycology: Open Access*.

[36] Lindsay K. L., Gibney E. R., McAuliffe F. M. (2012). Maternal nutrition among women from Sub-Saharan Africa, with a focus on Nigeria, and potential implications for pregnancy outcomes among immigrant populations in developed countries. *Journal of Human Nutrition and Dietetics*.

[37] Weatherspoon D. D., Miller S., Ngabitsinze J. C., Weatherspoon L. J., Oehmke J. F. (2019). Stunting, food security, markets and food policy in Rwanda. *BMC Public Health*.

[38] Krause H., Faße A., Grote U. (2019). Welfare and food security effects of commercializing African indigenous vegetables in Kenya. *Cogent food & agriculture*.

[39] Onyango A. O. M. (2010). *African Indigenous Vegetables in Kenya: Strategic Repositioning in the Horticultural Sector Volume 2 of Inaugural Lecture (Issue April 2010)*.

[40] Franz C. M. A. P., Huch M., Mathara J. M. (2014). African fermented foods and probiotics. *International Journal of Food Microbiology*.

[41] Gabaza M., Muchuweti M., Vandamme P., Raes K. (2017). Can fermentation be used as a sustainable strategy to reduce iron and zinc binders in traditional African fermented cereal porridges or gruels?. *Food Reviews International*.

[42] Umuhozariho M. G., Shayo N. B., Msuya J. M., Sallah P. Y. K. (2011). Utilization of cassava leaves as a vegetable in Rwanda. *Rwanda Journal*.

[43] Şanlier N., Gökcen B. B., Sezgin C. (2019). Health benefits of fermented foods. *Critical Reviews in Food Science and Nutrition*.

[44] Oluwafemi A. A. (2020). African sorghum-based fermented foods: past, current and future prospects. *Nutrients*.

[45] Wafula E. N. (2017). Effects of postharvest-processing technologies on the safety and quality of African indigenous leafy vegetables. https://ediss.sub.uni-Hamburg.de/handle/ediss/7392.

[46] Kamga R. T., Kouame C., Atangana A. R., Chagomoka T., Ndango R. (2013). Nutritional evaluation of five African indigenous vegetables. *Journal of Horticultural Research*.

[47] Kansiime M. K., Ochieng J., Kessy R., Karanja D., Romney D., Afari-Sefa V. (2018). Changing knowledge and perceptions of African indigenous vegetables: the role of community-based nutritional outreach. *Development in Practice*.

[48] Keatinge J. D. H., Wang J. F., Dinssa F. F. (2015). Indigenous vegetables worldwide: their importance and future development. *Acta horticulture*.

[49] Kwenin W. K. J., Wolli M., Dzomeku B. M. (2011). Assessing the nutritional value of some African indigenous green leafy vegetables in Ghana. *Journal of Animal & Plant Sciences*.

[50] Matenge S. T. P., Li J., Apau S., Tapera R. (2017). Nutritional and phytochemical content of indigenous leafy vegetables consumed in Botswana. *Frontiers In Food & Nutrition Research*.

[51] Mugwaneza O. (2018). *Indigenous Vegetable Production and the Economic Empowerment of Rural Women in Africa: Reality, Prospects, and Challenges in Rwanda*.

[52] Onyango C. M., Kunyanga C. N., Ontita E. G., Narla R. D., Kimenju J. W. (2013). Current status on production and utilization of spider plant (Cleome gynandra l.) an underutilized leafy vegetable in Kenya. *Genetic resources and crop evolution*.

[53] Shackleton C. M., Pasquini M. W., Drescher A. W. (2009). African indigenous vegetables in urban agriculture.

[54] Tufts H. R., Harris C. S., Bukania Z. N., Johns T. (2015). Antioxidant and anti-inflammatory activities of Kenyan leafy green vegetables, wild fruits, and medicinal plants with potential relevance for kwashiorkor. *Evidence-based Complementary and Alternative Medicine*.

[55] Van Zonneveld M., Kindt R., Solberg S., N’danikou S., Dawson I. K. (2021). Diversity and conservation of traditional African vegetables: priorities for action. *Diversity and Distributions*.

[56] Popova A., Mihaylova D. (2019). Antinutrients in plant-based foods: a review. *The Open Biotechnology Journal*.

[57] Ontita E., Onyango C., Onwonga R., Nyamongo D. (2016). Indigenous knowledge on the uses of African nightshades (Solanum nigram l.) species among three Kenyan communities. *Asian Journal of Agricultural Extension, Economics & Sociology*.

[58] Moyo M., Amoo S. O., Ncube B., Ndhlala A. R., Finnie J. F., Van Staden J. (2012). Phytochemical and antioxidant properties of unconventional leafy vegetables consumed in Southern Africa. *South African Journal of Botany*.

[59] Sivakumar D., Chen L., Sultanbawa Y. (2018). A comprehensive review on beneficial dietary phytochemicals in common traditional Southern African leafy vegetables. *Food Science and Nutrition*.

[60] Ochieng J., Afari-Sefa V., Karanja D., Kessy R., Rajendran S., Samali S. (2018). How promoting consumption of traditional African vegetables affects household nutrition security in Tanzania. *Renewable Agriculture and Food Systems*.

[61] Ogundola A. F., Bvenura C., Afolayan A. J. (2018). Nutrient and antinutrient compositions and heavy metal uptake and accumulation in S. nigrum cultivated on different soil types. *Scientific World Journal*.

[62] Filipiak S. A., Kurzawa M., Szłyk E. (2016). Simultaneous determination of selected anti-nutritional components in Asiatic plants using ion chromatography. *European Food Research and Technology*.

[63] Parkouda C., Ba/Hama F., Ouattara/Songre L., Tano-Debrah K., Diawara B. (2015). Biochemical changes associated with the fermentation of baobab seeds in Maari: an alkaline fermented seeds condiment from Western Africa. *Journal of Ethnic Foods*.

[64] Capozzi V., Fragasso M., Romaniello R., Berbegal C., Russo P., Spano G. (2017). Spontaneous food fermentations and potential risks for human health. *Fermentation*.

[65] Medina-Pradas E., Pérez-Díaz I. M., Garrido-Fernández A., Arroyo-López F. N. (2017). Review of vegetable fermentations with particular emphasis on processing modifications, microbial ecology, and spoilage. *The Microbiological Quality of Food: Foodborne Spoilers*.

[66] Yang X., Zhou J., Fan L., Qin Z., Chen Q., Zhao L. (2018). Antioxidant properties of a vegetable–fruit beverage fermented with two Lactobacillus plantarum strains. *Food Science and Biotechnology*.

[67] Diaz M., Kellingray L., Akinyemi N. (2019). Comparison of the microbial composition of African fermented foods using amplicon sequencing. *Scientific Reports*.

[68] Di Cagno R., Filannino P., Gobbetti M. (2015). Fermented foods: fermented vegetables and other products. *Encyclopedia of Food and Health*.

[69] Mokoena M. P., Mutanda T., Olaniran A. O. (2016). Perspectives on the probiotic potential of lactic acid bacteria from African traditional fermented foods and beverages. *Food & Nutrition Research*.

[70] Swain M. R., Anandharaj M., Ray R. C., Parveen Rani R. (2014). Fermented fruits and vegetables of Asia: a potential source of probiotics. *Biotechnology Research International*.

[71] Ivanova N., Gugleva V., Dobreva M., Pehlivanov I., Stefanov S., Andonova V. (2016). *We Are Intechopen, the World’s Leading Publisher of Open Access Books Built by Scientists, for Scientists Top 1%*.

[72] Felix A., Francis A. (2019). Effect of traditional fermentation process on the nutrient and anti-nutrient content of maize and African locust beans. *Journal of Food Science and Nutrition Research*.

[73] Johansen P. G., Owusu-Kwarteng J., Parkouda C., Padonou S. W., Jespersen L. (2019). Occurrence and importance of yeasts in indigenous fermented food and beverages produced in sub-Saharan Africa. *Frontiers in Microbiology*.

[74] Wafula E. N., Franz C. M. A. P., Rohn S. (2015). Fermentation of African leafy vegetables to lower post-harvest losses, maintain quality and increase product safety. Tropentag: management of land use systems for enhanced food security : Conflicts, controversies and resolutions. *African Journal of Horticultural Science*.

[75] Marshall E., Mejia D. (2012). *Traditional Fermented Food and Beverages for Improved Livelihoods*.

[76] Hasan M. N., Sultan M. Z., Mar-E-Um M. (2014). Significance of fermented food in nutrition and food science. *Journal of Scientific Research*.

[77] Chelule P. K., Madiba S., Mokgatle M. (2020). Perceptions and usage of selected fermented foods for feeding children aged 13-60 months in Tshwane, Gauteng province. *Pan African Medical Journal*.

[78] Adebo O. A., Medina-Meza I. G. (2020). Impact of fermentation on the phenolic compounds and antioxidant activity of whole cereal grains: a mini review. *Molecules*.

[79] Chen C., Zhao S., Hao G., Yu H., Tian H., Zhao G. (2017). Role of lactic acid bacteria on the yogurt flavour: a review. *International Journal of Food Properties*.

[80] Karenzi E., Mashaku A., Nshimiyimana A. M., Munyanganizi B., Thonart P. (2013). Kivuguto traditional fermented milk and the dairy industry in Rwanda. A review. *Biotechnologie, Agronomie, Société et Environnement = Biotechnology, Agronomy, Society and Environment [=Base]*.

